# A Corpus-Based Study of Cohesion in English Medical Texts and its Chinese Translation

**Published:** 2009-09

**Authors:** Jia Zhao, Wenli Yan, Yumei Zhou

**Affiliations:** *Department of Foreign Languages, the Fourth Military Medical University, Xi’an, China*

**Keywords:** cohesion, cohesive device, medical text, translation

## Abstract

Cohesion as an indispensable linguistic feature in discourse analysis and translation has aroused many researchers’ interest. To explore the regularity in shifting cohesive devices from English into Chinese our study was designed to analyze the similarities and differences of cohesive devices between English medical texts (EMTs) and their Chinese translation texts (CTTs). A parallel corpus consisting of 15 EMTs and 15 CTTs was established, each type of cohesive devices was identified, and paired *t*-test was run for statistic analysis. We have revealed that both EMTs and CTTS share more similarities than differences in the use of cohesive devices; the differences between them only exist in the employment of reference in terms of occurrence frequencies; the majority of cohesive devices are maintained in Chinese translation for precision, clarity and logicality. Our study will not only help medical students and medical workers but also shed light on EMP teaching and research.

## INTRODUCTION

In 1960s the emergence of text linguistics overcame the limitation of sentence-oriented study and elevated the study of language from sentence level to textual level. When text linguistics was introduced into the study of translation, the basic communicative units in translation were shifted from words or isolated sentences to texts. Cohesion as “visible network” of a text plays a significant role in organizing linguistic elements into a unified whole text and naturally becomes one of the most important subjects of text translation.

With the development of contrastive linguistics and text translation, many researchers have conducted comparative studies of cohesive devices in different text types between English and Chinese and have analyzed the role of cohesion in translation. However, few studies have been reported on a comparative study of cohesion in English and Chinese medical texts until now. Based on cohesion theory proposed by Halliday and Hason (1), our study was designed to analyze the similarities and differences of cohesive devices in English medical texts and their Chinese translations in textbooks and to explore the regularity in shifting cohesive devices from English into Chinese.

## METHODS

### Pretest

In order to attest the feasibility of our study, a pretest was conducted, in which a mini-sized parallel corpus was established with five English medical texts and their Chinese versions. The sample texts in the corpus were randomly selected from two English medical textbooks: *Subject-based English: English for Medicine (Book 1 & Book 2)* which were published by Foreign Language Teaching and Research Press in China. The occurrence frequencies of cohesive devices used in the sample texts were firstly hand-tagged and then computer-analyzed statistically. The results showed some significant differences of cohesive devices in English and Chinese medical texts.

### Establishment of a parallel corpus

**Selection of textbooks for a parallel corpus.** Five English medical textbooks were selected according to the criteria proposed by Nwogu (2): representativity, reputation, and accessibility. They were:

- *New Century Medical English Course: Biological Medicine (Student's Book);*
- *New Century Medical English Course: Biological Medicine (Teacher's Book);*
- *Subject-based English: English for Medicine* 1;
- *Subject-based English: English for Medicine* 2;
- *A New Course Book for English in Military Studies: Military Medicine.*

In terms of representativity, the selected textbooks belong to medical branch of the professional English textbook and include five disciplines of medicine: clinical medicine, basic medicine, public health, pharmacology and military medicine.

In terms of reputation, the first four medical textbooks are recommended as foreign language textbooks for medical college students by Ministry of Education of China. The fifth one is a new century textbook specifically designed for military academies and universities. All of them were published by Foreign Language Teaching and Research Press or Shanghai Foreign Language Education Press, the two most prestigious and professional foreign language publishing houses in China.

In terms of accessibility, the selected medical textbooks are available in many bookstores around China or accessible from their publishing presses.

In addition, English texts in these textbooks are adapted from “original materials” which were written by native speakers of English to keep authenticity of English in use, and their Chinese counterparts have been translated by the medical professionals who have rich experience in medical translation.

**Selection of sample texts for the parallel corpus.** Totally 30 sample texts including 15 English medical texts and 15 parallel Chinese translations were selected from the five textbooks for the parallel corpus.

The sample texts were selected in accordance with two criteria: 1) the selected EMTs are derived from five disciplines of medicine: clinical medicine, basic medicine, public health, pharmacology and military medicine; 2) the length of each selected EMT is about 1000 ∼ 2500 running words.

Based on the criteria, we conducted a two-round stratified sampling in choosing the medical texts for the corpus. In the first round, all the EMTs which met our criteria were selected and numbered. A total of 92 English medical sample texts were randomly selected from all the texts in the five textbooks: 26 in basic medicine, 33 in clinical medicine, 8 in public health, 3 in pharmacology and 22 in military medicine. In the second round, 3 texts in each medical discipline were randomly selected from 92 texts and made up an English corpus and their parallel Chinese versions constituted the Chinese corpus. Thus, a parallel corpus of English and Chinese medical texts was established.

### Data collection and analysis

Halliday and Hason define five types of cohesive devices in their book *Cohesion in English*, which are reference, substitution, ellipsis, conjunction and lexical cohesion. In our study cohesive devices in EMTs and CTTs were identified based on their classification. Cohesive devices between sentences stand out more clearly as they are “the only source of texture”. Therefore, it is the intersentence cohesion that is significant because it represents the variable aspect of cohesion, distinguishing one text from another. Accordingly, the present study was focused on cohesion across sentence boundaries.

**Procedures of analysis.** For each sample text in the parallel corpus, we made an analysis in the following procedures: firstly, numbering each sentence in English medical texts and their Chinese translations; secondly, identifying the cohesive ties and types they belong to; finally, counting the total number of each type of cohesive ties in each sample text to obtain the total number of cohesive ties of each type in EMTs and CTTs.

**Samples of analysis.** To describe the process of identification in a more detailed way, we randomly selected a paragraph in a sample text from the EMTs and its corresponding Chinese version as an example, in which the underlined items were the presupposed elements and the darkened items were the presupposing elements or cohesive ties.

1) *The term radiosensitivity describes the inherent properties of a tumor that determine its responsiveness to radiation.* 2) *It varies widely among the different types of cancers.* 3) *For example, lymphomas are highly radiosensitive, whereas rhabdomyosarcomas and melanomas are much less so.* 4) *The radiation dose that is chosen for treatment of a particular cancer is determined by factors such as the radiosensitivity of the tumor type, size of tumor, and, more importantly, the tolerance of the surrounding tissues.* 5) *The ability to give graded, fractional doses of radiation and quantitate the number of cells surviving permits the development of a dose-response curve.*

In sentence 1), there were only two cohesive ties “tumor” and “radiation”. They referred back to the same items in the preceding paragraph. Such repetition is a typical phenomenon in the corpus. In this sentence the first and second anaphoric “the”, as a forward reference, were limited to the structure type. Unlike the selective demonstratives (*this, these* and *here*), the can never refer forward cohesively and it can only refer to a modifying element within the same nominal group as itself. Although “its” in this sentence referred to “tumor” and had the function of reference, the two items were intrasentence and belonged to structure cohesion.

In sentence 2), there were two types of cohesive devices: reference and collocation. “It” in the sentence referred to underlined “radiosensitivity”, and the lexical item “cancers” co-occurred with underlined “tumor” in sentence 1).

Five cohesive ties were identified in sentence 3). The conjunctive item “for example” was used to illustrate the phenomenon described in sentence 2), thus it linked these two sentences closely. “radiosensitive” as repetition referred back to the lexical item “radiosensitivity” in sentence 1) since a lexical item is not bound to a particular grammatical category or a particular morphological form. The other three cohesive devices “lymphomas”, “rhabdomyosarcomas” and “melanomas” were hyponyms of the underlined “tumor” in sentence 1). The conjunctive device “whereas” as structure cohesion did not play a cohesive role of adversative in the same sentence. According to Halliday, the meaning of a conjunctive item creates a specific relationship between the two sentences before and after this item.

In sentence 4), there were seven cohesive ties of repetition and one collocation. The items of “radiation dose”, “type” and “surrounding tissues” which appeared in the preceding paragraphs were employed again, and the items of “tumor”, “cancer” and “radiosensitivity” referred back to the same items which occurred in the first three sentences respectively. The item “treatment” collocated with “cancers” in sentence 2). Much attention should be paid to the comparative adverb “more importantly” in this sentence. As an adjunct in the clause it did not play a cohesive role because the cohesive relation of comparative reference can only be created between sentences.

In sentence 5), the phrase “doses of radiation” which was the repetition of the lexical item “radiation dose” in sentence 4) was the only cohesive tie.

In the English paragraph, we identified a total of 18 cohesive ties including 1 reference, 1 conjunction and 16 lexical cohesions. The one reference was the personal reference “it” and the one conjunction was the additive “for example”. The 16 lexical cohesions were subcategorized into 14 reiterations and 2 collocations of “cancers/tumor” and “treatment/cancers”. In the 14 reiterations, 11 were repetitions, 3 were hyponyms such as “lymphomas - tumor”, “rhabdomyosarcomas - tumor” and “melanomas - tumor”.

In sum, the number of cohesive ties, specific items and types in the above English paragraph is clearly showed in Table 1.

**Table 1:** Number of cohesive ties, specific items and types in the English paragraph

Sentence No.	No. of cohesive ties	Cohesive item	Type

1	2	Tumor	reiteration (L)
		Radiation	reiteration (L)
2	2	It	personal (R)
		cancers/tumor	collocation (L)
3	5	For example	additive (C)
		lymphomas-tumor	reiteration (L)
		Radiosensitive	reiteration (L)
		rhabdomyosarcomas-tumor	reiteration (L)
		melanomas-tumor	reiteration (L)
4	8	radiation dose	reiteration (L)
		treatment/cancers	collocation (L)
		Type	reiteration (L)
		Cancer	reiteration (L)
		Radiosensitivity	reiteration (L)
		tumor × 2	reiteration (L)
		surrounding tissues	reiteration (L)
5	1	doses of radiation	reiteration (L)
Total	18		

“-”, Connecting hyponyms; “/”, Linking collocations; “×”, Indicating times.

In the above English paragraph, we identified a total of 18 cohesive ties including 1 reference, 1 conjunction and 16 lexical cohesions. The one reference was the personal reference “it” and the one conjunction was the additive “for example”. The 16 lexical cohesions were subcategorized into 14 reiterations and 2 collocations (“cancers/tumor” and “treatment/cancers”). In the 14 reiterations, 11 were repetitions, 3 were hyponyms such as “lymphomas - tumor”, “rhabdomyosarcomas - tumor” and “melanomas - tumor”.

The following is the parallel Chinese translation of this English paragraph.


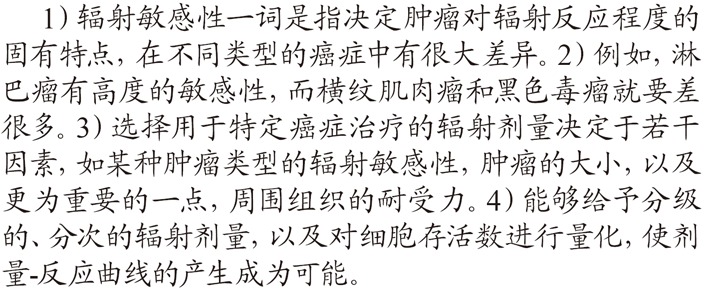


In the same way the number of cohesive ties, specific items and types of the Chinese version were analyzed and indicated in Table 2.

**Table 2:** Number of cohesive ties, specific items and types in the Chinese paragraph

Sentence No.	No. of cohesive ties	Cohesive item	Type

1	3	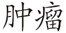	reiteration (L)
		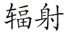	reiteration (L)
		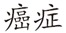	reiteration (L)
2	5	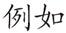	additive (C)
		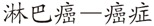	reiteration (L)
		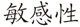	reiteration (L)
		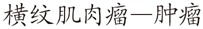	reiteration (L)
		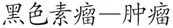	reiteration (L)
3	7	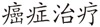	reiteration (L)
		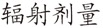	reiteration (L)
		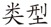	reiteration (L)
		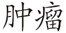 × 2	reiteration (L)
		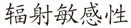	reiteration (L)
		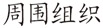	reiteration (L)
4	1	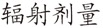	reiteration (L)
Total	16		

“×”, Indicating times.

A total number of 16 cohesive ties were identified in the above Chinese paragraph. The personal reference “it” in English sentence 2) was omitted in Chinese to avoid verbosity. The two cohesive ties of “treatment” and “cancer” in sentence 4) were translated into one nominal phrase “
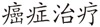
” which referred back to the same item in the preceding sentence. Among the 16 cohesive ties, there was only one conjunction 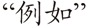 and the rest were reiterations including 13 repetitions and 3 hyponyms of “
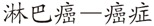
”, “
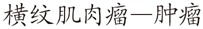
” and “
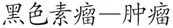
”.

### Data analysis

All the identified cohesive devices in the sample texts were categorized and the occurrence frequencies of cohesive devices in medical texts of the parallel corpus were counted manually, added up and recorded. The total number of each type of cohesive ties in EMTs and CTTs was compared. Paired *t*-test with SPSS13.0 software was run for the statistical analysis of cohesive devices and the Alpha value was set up at p<0.05.

## RESULTS AND DISCUSSION

### Distribution and comparison of overall cohesive devices in EMTs and CTTs

As shown in Table 3, EMTs yielded a total of 2,796 cohesive ties in four types of cohesive devices with no occurrence of ellipsis, whereas CTTs showed a total of 2,800 cohesive ties in four types without substitution. The cohesive devices in both EMTs and CTTs followed the same decreasing order in terms of their occurrence frequencies. In parallel corpus, lexical cohesion was the overwhelmingly used device (87.5% in EMTs, 89.4% in CTTs), followed by reference (8.9% in EMTs, 7.1% in CTTs) and conjunction (3.5% in EMTs, 3.46% in CTTs). Ellipsis (0% in EMTs, 0.04% in CTTs) and substitution (0.1% in EMT, 0% in CTT) occurred with extremely low frequencies. Lexical cohesion, reference and conjunction are consistently the three frequently used devices in the parallel corpus. The distribution of cohesive devices manifested some similarities in the preference of cohesive types. No significant difference was found in the overall cohesive devices in terms of their occurrence frequencies between EMTs and CTTs (p>0.05). On the whole, cohesive devices were employed in EMTs as frequently as in CTTs.

**Table 3:** Frequency and percentage of five types of cohesive devices

Corpus	EMTs	CTTs

Cohesive devices	Frequency (%)	Frequency (%)

Reference	249 (8.9)	199 (7.1)
Substitution	4 (0.1)	0 (0)
Ellipsis	0 (0)	1 (0.04)
Conjunction	97 (3.5)	97 (3.46)
Lexical cohesion	2446 (87.5)	2503 (89.4)
Total	2796	2800

A medical text as an information-oriented expository text aims at presenting facts and providing readers with a large amount of information. Since the linear organization of text follows the clustering of information, lexical cohesion built upon semantic relations reflects clusters. Therefore, lexical cohesion plays a pivotal role in structuring a medical text and making it coherent as a whole. Furthermore, as an academic text, a medical text is required to be accurate and precise. The occurrence of substitution and ellipsis often used to set up cohesive relation between sentences in a conversation or dialogue is seldom used in a medical text, which may cause some ambiguity. Therefore, little attention was devoted to the similarity and difference of substitution and ellipsis in our study.

### Distribution and comparison of lexical device in EMTs and CTTs

We found from Table 4 reiteration (93.0% in EMTs, 92.8% in CTTs) was the dominant device of lexical cohesion in EMTs and CTTs in terms of occurrence frequencies while collocation (7.0% in EMTs, 6.2% in CTTs) showed a very low frequency. No significant differences existed in the occurrence frequencies of the overall devices of lexical cohesion and its subcategories between EMTs and CTTs (p>0.05).

**Table 4:** Frequency and percentage of subcategories of lexical cohesion

Corpus	EMTs	CTTs

Cohesive devices	Frequency (%)	Frequency (%)

Reiteration	2276 (93.0)	2322 (92.8)
Collocation	170 (7.0)	181 (6.2)
Total	2446	2503

Reiteration as the most frequently used device contributes greatly to the cohesion of medical text as a whole. From the perspective of communication, lexical items related to the main topic need to be repeated for emphasis now and then since the information every sentence conveyed cannot be totally new. As for collocation, it is specifically associated with some particular medical register or functional variety of language. Although collocation in EMTs and CTTs is much less employed than reiteration, it substantially contributes to the cohesion of medical texts because it extends the boundaries of sentences and even paragraphs to bind the whole text.

As a prominent cohesive device, reiteration can be divided into four subcategories: repetition, synonym, superordinate and “general” item. Table 5 illustrated the occurrence frequencies and percentages of the four subcategories in EMTs and CTTs.

According to Table 5, repetition (81.1% in EMTs, 81.14% in CTTs) displayed the highest percentage of lexical cohesion, followed by synonym (16.4% in EMTs, 17.14% in CTTs), superordinate (1.8% in EMTs, 1.68% in CTTs) and “general” item (0.7% in EMTs, 0.04% in CTTs). No significant statistical difference was observed in the occurrence frequencies of subcategories of reiteration except “general” item (p>0.05).

**Table 5:** Frequency and percentage of subcategories of reiteration

Corpus	EMTs	CTTs

Cohesive devices	Frequency (%)	Frequency (%)

Repetition	1846 (81.1)	1884 (81.14)
Synonym	373 (16.4)	398 (17.14)
Superordinate	42 (1.8)	39 (1.68)
“General” item	15 (0.7)	1 (0.04)
Total	2276	2322

As is mentioned by Wright (3), lexical explicitness, the most striking feature of a text depends on lexical cohesion, especially repetition rather than reference such as pronouns. As for synonym, according to Halliday, if two or more lexical items carry the same or nearly the same ideational meaning, they can be defined as synonym regardless of their parts of speech. Since synonym is an indispensable lexical device, semantic relationships between lexical items of different parts of speech are built up through it. However, it is less frequently used than repetition because more attention is paid to accuracy and clarity rather than the variety of expression in a medical text. The use of superordinates in medical texts is closely related to the specific fields of medicine. The occurrences of these words and their hyponyms or meronyms provide some semantic references to each other and function dependently as a network to contribute to the integration of medical texts. Although “general item” shows a fairly low proportion in EMTs and CTTs, it still facilitates the realization of cohesion throughout texts.

Repetition, synonym and superordinate are used in EMTs as frequently as in their Chinese translations because of the informative function and stylistic features of medical texts. Repetition enjoys the highest frequency in parallel corpus for it is claimed to be the most pervasive device in scientific literature to emphasize the correctness of concept (4) and to give prominence to topic. Synonym is another important device to create cohesion in EMTs and CTTs. Almost every pair of synonyms in Chinese can match their equivalent expressions in English and vice versa because both languages have their own abundant lexical items of synonyms and near-synonyms. Superordinate in medical texts occurs regularly when an object is classified or the internal structure of an entity is described. No difference was found in the expression of superordinate between EMTs and CTTs (p>0.05).

As for “general” item, according to Halliday, it is worth stressing that general words are very general in meaning so they are often interpretable only by reference to some element other than themselves. They require recourse to another item that must be located earlier within the same text. No matter in English or Chinese “general” item plays a significant role in making a text integrated as a whole. The statistical result of “general” item showed significant difference in occurrence frequencies between EMTs and CTTs (p<0.05). That is to say, “general” items are more frequently used in EMTs than in CTTs, and such discrepancy is due to the fact that the presupposing general words are replaced by the presupposed elements which are appeared in the preceding part of the text in E-C translation for the sake of clarity and accuracy.

Although collocation differs greatly in English and Chinese languages, its discrepancy is scarcely showed between English medical texts and their Chinese versions as the collocational relationships between lexical items in a medical text are established for referential function. For example:

*The global importance of food safety is not fully appreciated by many public health authorities. Epidemiological surveillance has demonstrated a constant increase in the prevalence of food-born illness.*


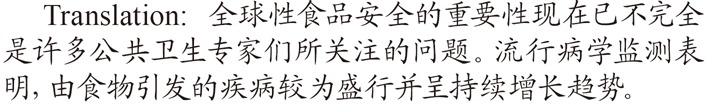


### Distribution and comparison of reference device in EMTs and CTTs

Among the three subcategories of reference in Table 6, demonstrative reference (62.7% in EMTs, 66.3% in CTTs) accounted for the largest proportion in parallel corpus, followed by personal reference (20.0% in EMTs, 17.1% in CTTs) and then comparative reference (17.3% in EMTs, 16.6% in CTTs) in a descending order.

**Table 6:** Frequency and percentage of subcategories of reference

Corpus	EMTs	CTTs

Cohesive devices	Frequency (%)	Frequency (%)

Demonstrative reference	156 (62.7)	132 (66.3)
Personal reference	50 (20.0)	34 (17.1)
Comparative reference	43 (17.3)	33 (16.6)
Total	249	199

Demonstrative reference is the most frequently used device in both EMTs and CTTs, which is attributed to the stylistic features of medical texts. Personal reference entirely depends on other item for its interpretation and has no definitional meaning in itself. The use of personals in sentences with intensively packed information might easily lead to confusion and ambiguity because it is difficult to identify the references of personals in various nouns.

Despite the same distribution tendency of the subcategories of reference, significant difference was found in occurrence frequencies of personal reference and demonstrative reference between EMTs and CTTs (p<0.05) but there was no significant difference in the statistical result of comparative reference (p>0.05). Larson (5) points out that “it is quite common in English to introduce a new participant with a noun phrase and then refer to this participant by a pronoun throughout the rest of the paragraph”. However, this is not the case in Chinese where personal or demonstrative pronouns are often omitted or substituted by a noun phrase that the pronoun presupposes. The absence of definite article the in Chinese contributes to the difference of demonstrative reference between EMTs and CTTs. For example:

*Cilia are hairlike appendages that actively beat back and forth, moving a layer of mucus away from the lungs. Particles and bacteria are trapped in the mucus layer, preventing them from reaching the delicate air-exchange membranes in the lung.*


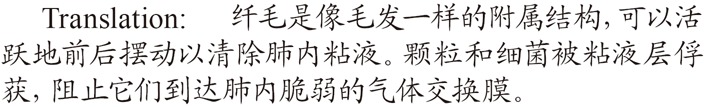


As the comparative referential system in Chinese bears great similarity to that in English, the comparative reference in both languages not only has the same basic concepts but also can be embodied by adjectives and adverbs, which may well explain why there was no significant difference in occurrence frequencies of comparative reference between EMTs and CTTs.

### Distribution and comparison of conjunction device in EMTs and CTTs

According to Table 7, the occurrence frequency of the subcategories of conjunction demonstrated that the most frequently used devices in the sample texts of the parallel corpus were additive device (39% in EMTs, 38% in CTTs) and adversative device (31% in EMTs, 30% in CTTs) whereas causal device (16% in EMTs, 18% in CTTs) and temporal device (14% in both EMTs and CTTs) were less frequently used.

**Table 7:** Frequency and percentage of subcategories of conjunction

Corpus	EMTs	CTTs

Cohesive devices	Frequency (%)	Frequency (%)

Additive	38 (39.0)	37 (38.0)
Adversative	30 (31.0)	29 (30.0)
Causal	16 (16.0)	17 (18.0)
Temporal	13 (14.0)	14 (14.0)
Total	97	97

The total percentages of additive and adversative device in parallel corpus (70% in EMTs, 68% in CTTs) accounted for over half proportion of total amount of conjunctive ties. The frequent employment of these two subcategories can be attributed to their functions in medical texts. Additives are used to illustrate the propositions, introduce examples, add information and substantiate ideas while adversatives are employed to draw conclusions, present or explain information and make contrasts. Causal and temporal device, though used not as much as adversatives and additives, have the function of deduction and succession.

There was no statistical difference of occurrence frequencies of conjunction and its four subcategories between the EMTs and CTTs (p>0.05). According to Yongsheng Zhu *et al* (6), semantic relation in Chinese is not explicitly showed by conjunctive ties but indicated by logic and sentence order implicitly. On the contrary, English emphasizes the explicit means to show semantic relations between sentences or paragraphs so conjunctions are highly employed. Since medical texts are consistent in logic and well knit in structure, conjunction device is frequently used in both EMTs and CTTs to show the logical relations between facts. For example:

*...third, improved access to technologies that people can use to prevent infection (eg, condoms); fourth, reduction of the stigma associated with STDs, and, finally, improved surveillance.*


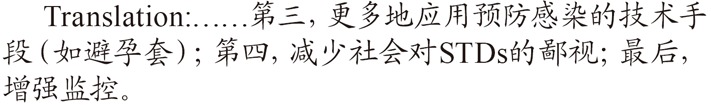


In the English sentence “third”, “fourth”, and “finally” clearly display the semantic relations of sequence. They are maintained in Chinese version with their equivalent expressions of 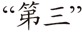, 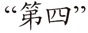 and 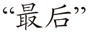 to avoid ambiguity and confusion.

## CONCLUSION

On the basis of quantitative and comparative analysis, we can draw the following conclusions:

Firstly, English medical texts and their Chinese translations share more similarities than differences in the use of cohesive devices. The similarities between them mainly exist in two aspects. The three mainly used cohesive types and their subcategories show the same distribution tendency in both EMTs and CTTs. Besides, the overall cohesive devices, conjunction device and lexical cohesion in EMTs are used as frequently as those in CTTs. These similarities can be attributed to the informative function and stylistic features of medical texts. As expository writing, medical texts are characteristic of objectivity, precision, explicitness and logicality.

Secondly, there are some differences between EMTs and CTTs in the use of specific devices of reference in terms of their occurrence frequencies. Demonstrative reference and personal reference are more frequently used in EMTs than in CTTs. Instead of using explicit cohesive markers in English, Chinese prefers the omission of reference and repetition of nominal nouns to create cohesion at textual level. Such discrepancy is mainly due to the specific features of the two languages: English is more hypotactic while Chinese is more paratactic.

Our study is a tentative investigation into cohesion in English and Chinese medical texts and is a new research in English and Chinese translation of medicine. Based on the establishment of a parallel corpus, we have analyzed the similarities and differences of cohesive devices between English medical texts and their Chinese translations. Since EMTs and CTTs bear great resemblance, the majority of cohesive devices can be maintained in E-C translation for the sake of preciseness, clarity and smoothness. In some cases, however, it is not an effective way to maintain the cohesive devices without changing their forms and meanings so it is necessary to employ some translation techniques to achieve the closest natural equivalence at the maximum level. The findings of our study may help medical students and medical workers have a better understanding of the regularity of the use of cohesive devices in English and Chinese medical texts, shed light on their practice of medical translation, and help them lay a solid foundation for the information rendering from the original text into the target text accurately and smoothly.
